# Robust and Efficient Indoor Localization Using Sparse Semantic Information from a Spherical Camera

**DOI:** 10.3390/s20154128

**Published:** 2020-07-24

**Authors:** Irem Uygur, Renato Miyagusuku, Sarthak Pathak, Alessandro Moro, Atsushi Yamashita, Hajime Asama

**Affiliations:** 1 Department of Precision Engineering, The University of Tokyo, 7-3-1 Hongo, Bunkyo-ku, Tokyo 113-8656, Japan; pathak@robot.t.u-tokyo.ac.jp (S.P.); alessandromoro.italy@ritecs.co.jp (A.M.); yamashita@robot.t.u-tokyo.ac.jp (A.Y.); asama@robot.t.u-tokyo.ac.jp (H.A.); 2 Department of Mechanical and Intelligent Engineering, Utsunomiya University, Utsunomiya 321-8585, Tochigi, Japan; miyagusuku@cc.utsunomiya-u.ac.jp

**Keywords:** semantic localization, indoor localization, crude maps

## Abstract

Self-localization enables a system to navigate and interact with its environment. In this study, we propose a novel sparse semantic self-localization approach for robust and efficient indoor localization. “Sparse semantic” refers to the detection of sparsely distributed objects such as doors and windows. We use sparse semantic information to self-localize on a human-readable 2D annotated map in the sensor model. Thus, compared to previous works using point clouds or other dense and large data structures, our work uses a small amount of sparse semantic information, which efficiently reduces uncertainty in real-time localization. Unlike complex 3D constructions, the annotated map required by our method can be easily prepared by marking the approximate centers of the annotated objects on a 2D map. Our approach is robust to the partial obstruction of views and geometrical errors on the map. The localization is performed using low-cost lightweight sensors, an inertial measurement unit and a spherical camera. We conducted experiments to show the feasibility and robustness of our approach.

## 1. Introduction

Self-localization—i.e., obtaining one’s pose information within a map—is necessary to navigate and interact with the environment. Localization methods have a wide range of applications, from monitoring robots to facilitating assistance systems. Thus, depending on the system, these methods face challenges in terms of factors such as achieving acceptable accuracy and robustness as well as reducing complexity and sensor costs. The outdoor localization issue is mostly solved using GPS. However, because GPS is not very effective inside buildings, indoor localization remains a challenge. In the absence of GPS systems, different information sources are required for indoor localization. Radio frequency ID (RFID) tags, ultrasonic tags and infrared emitters are potentially viable alternatives; however, these methods require prior installation and modifications to the infrastructure [1,2,3].

Another option is to exploit already available infrastructure (without modification). Range finders and cameras are commonly used with simultaneous localization and mapping (SLAM) and Monte Carlo localization (MCL) for robotics applications. For systems that require high accuracy, weight and power requirements—and where these can be afforded—dense range scans provided by expensive sensors, such as LiDARs, can be an optimal choice. However, for small robots or assistance systems designed to be carried by people, light and inexpensive sensors need to be selected, at the cost of their poor performance. Vision-based systems enable the use of lightweight low-cost cameras for localization.

Visual SLAM (VSLAM) uses visual information to construct the map of an environment while simultaneously self-localizing within it. Although pose estimates are globally consistent within the constructed map, there is no guarantee that the results are globally consistent with the real world due to various factors such as dynamic environments, drifts, scaling and repeated patterns [4]. Globally consistent and human-readable 2D floor maps already exist for most indoor environments. Thus, instead of constructing a map of an indoor environment and confronting possible consistency problems, an already existing indoor map can be used as the source for prior information for the MCL.

A vision-based MCL (VMCL) uses the information obtained from a camera to relate to an a priori environment map via multi-modal distributions to localize the user globally [5,6,7]. To make it easier and more user-friendly to obtain a geometrically accurate map, some methods use already available blueprints or 2D floor maps as a base to build their feature or point cloud maps; they extrapolate the 2D maps to 3D and map the image features. Winterhalter et al. [8] constructed a point cloud from RGB-D distance measurements to match a 2D floor map, which was extrapolated to a 3D map by adding an extra dimension for the ceiling height under the Manhattan world assumption. Chu et al. [9] replaced the RGB-D sensor with a smartphone camera and converted a 2D floor plan into 3D piecewise floor models to match the constructed point clouds, lines and free space.

However, there are some issues with these methods. Neither point clouds nor feature points are easy to calculate. Furthermore, creating these maps might be difficult and time-consuming. Moreover, after the careful initial preparation, these maps need to be updated against changes, which might occur from the replacement of objects. Such scenarios might require the repetition of the initial preparation process. Therefore, it should be easy for regular users to prepare and update these maps; moreover, the localization method should be robust to minor map errors.

Another problem facing this approach is that most buildings consist of ceilings, walls and floors, which are non-descriptive. Furthermore, vision-based systems might fail in cases in which feature points cannot be computed due to a blocked view caused by the system facing a wall, corner or an object. To solve the blocked view problem, cameras with a large field of view (FOV) have been used for indoor localization using different techniques, such as matching 2D lines extracted from a spherical image to a line descriptor map under the Manhattan world assumption [10] and matching scale-invariant feature transform (SIFT) features to a built image database [11]. Although these approaches are highly accurate and can manage possible view blockages, the amount of computation and storage required for matching large amounts of 3D point clouds or feature points can be too demanding for a small mobile or portable system with limited resources. Heavy computation and storage problems are generally caused by the use of distance measurements and feature points, which need to be obtained in considerable amounts to represent the environment, as observing a single feature point or a 3D point provides little additional data regarding localization. Conversely, obtaining semantic information from a single object, such as desks and doors, provides greater information than feature points and has become easier with the advent of convolutional neural networks (CNNs). Recent improvements in computer vision have made it possible to obtain human-level semantic information and are being incorporated into localization systems. The ability to classify objects creates a non-geometric distinction in the environment, which decreases entropy, even with a small amount of information. Therefore, it can be used as input to create efficient localization systems that can resolve the aforementioned problems. In this study, we refer to object classes as semantic information and sparsely distributed objects in the environment as sparse semantic information. We propose a sparse semantic localization method with the following features.

- It is based on the idea that semantic information, even in sparse amounts, provides more information gain, reduces uncertainty more efficiently than other input formats and can be used directly in the sensor model for efficient localization.
- It uses a large-FOV camera that is robust to the blocked view problem in indoor environments, where the users generally move close to objects and walls.
- It uses a minimal human-level data representation such that, given a 2D blueprint of the environment, users can easily prepare annotated maps by simply marking the approximate object centers on the blueprint. The localization method is robust to possible geometrical errors arising from approximations and minor mistakes during map preparation.

**Figure 1:**
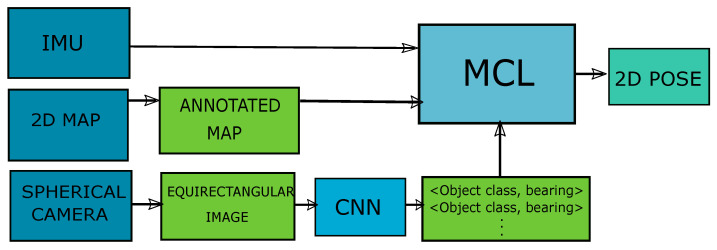
System overview: The proposed localization system using a 2D annotated map, equirectangular images from a spherical camera and an inertial measurement unit (IMU) carried by the user to self-localize. Images are processed using a convolutional neural network (CNN) to obtain the object class and bearing angle to the object center tuple for each object.

We used low-cost sensors to achieve a fast, robust and efficient localization system to obtain a 2D pose (x,y,θ) on a 2D map. We believe such a method would be particularly suitable for small systems with limited sources. The system overview is shown in Figure 1. We believe it is important for our system to be robust to the blocked view problem and geometric map errors. Therefore, to demonstrate the feasibility and robustness of our system, experiments with different FOVs and geometric map errors have been conducted in real life. The effect of different object classes on the localization system—and by extension, our system—has also been evaluated. To assess the robustness of our system against localization failures, pose errors were manually introduced to evaluate the system behavior in such cases. The performance of the sensor model in a symmetrical environment was also evaluated.

## 2. Related Works

Traditionally, robotic tasks, such as localization and navigation, are performed using geometric data with geometric maps and feature points with feature maps. Besides these traditional representations, interactive systems for human–robot cooperation tasks and assistant systems also need to have the conceptual information of the environment, such as object classes, to be able to interact with humans and the human world.

Because geometric or feature point maps cannot provide conceptual knowledge regarding the environment, localization and mapping systems have begun to incorporate a semantic map in addition to the geometric or feature map [12]. VSLAM methods have begun to use semantic information with feature points or geometric maps to achieve loop closure [13], map sparsification and traceability [14] and bundle adjustment [15]; they also have begun to filter outliers to correctly map dynamic environments [16]. Although these mapping methods can be effective, combining geometric information or feature points and semantic information requires extra map registration and calibration. Furthermore, VSLAM methods generally lack global consistency and tend to drift.

Different localization systems with globally consistent prior maps have also started utilizing semantic information. Atanasov et al. [17] focused on the data association problem and incorporated semantic observations into the metric optimization via a set-based Bayes filter. Gawel et al. [18] developed a localization method under different viewpoints through graph extraction from semantic input and graph matching using graph descriptors. The MCL approaches also have begun to use semantic information, not only as a single landmark to localize around but also as a sensor model input. A system with an RGB-D camera was localized in a changing warehouse environment by combining the beam model using distance and bearing angle measurements and an a priori correlation table for objects [19]. The measurements were matched to an annotated grid map. Another work achieved a memory-efficient semantic localization of a car by matching semantic observations to a semantic feature map, in which each point had a 3D position and an object class, and the effectiveness of the semantic features and that of the SIFT features for localization were compared. Although the semantic map occupies three times less space than the SIFT map, the authors still used a very dense map for matching. Pöschmann et al. [20] converted an annotated 2D floor map into a 3D point cloud to synthesize images for pixel-wise comparison to real-world panoramic images for localization. Although the researchers aimed at localization, which they achieved using building structure classes, their method suffered from a high computational cost and failure concerning the furniture object classes. Mendez et al. [21] combined beam and likelihood models for semantic localization using an RGB-D camera, a 2D annotated floor map and a trained CNN performing pixel-wise segmentation on RGB images. For the likelihood model, a Look-Up Table (LUT) similar to the Chamfer distance was prepared for each object class. The authors expressed a preference for the combination of beam and likelihood models because of the smoothness provided by the gradient between the cells in the likelihood model and avoided possible measurement errors arising from ray casting such as incorrect measurements of an open door.

Similar to these previous works, we believe in the definition power of semantics and the effectiveness of working with smaller amounts of semantic information, instead of large point clouds or feature points, to achieve a localization system with computation and storage. We also believe in more direct solutions for smaller systems that might lack resources. Instead of using distance measurements, LUTs or the combination of likelihood and beam models, we propose the use of only a relative bearing angle to detect the object center and the object class names as sensor inputs; we also propose that each object class should be treated as a different sensor with different weights for indoor localization. Unlike many vision-based methods that require a certain overlap between consecutive frames to match feature points or dense scan methods, our sensor model requires only an approximate estimate of object centers and object labels. Therefore, it is not dependent on an object’s appearance for the localization process. The relative bearing angles are calculated from the user to the centers of the detected object’s bounding boxes. The bounding boxes and the object class names are obtained using a CNN that detects semantic bounding boxes to achieve real-time results. Because of the use of the relative bearing angles, we can say that the distribution of the detected object labels from different classes is used for localization. Although this method might seem similar to the methods using the relative bearing angle to localize around unique landmarks with known positions [22,23], we did not attempt to localize around any unique landmark. As a matter of fact, we did not have unique landmarks or data associations; we had objects from the same or different classes in the environment whose object class detection and bearing angles to the centers were used as the sensor input for the localization algorithm. In place of feature descriptors or dense scans, we relied on the distinction power of the sparse semantic information. Our method required a 2D annotated map, which is a 2D floor map with annotated objects. For each annotated object, the object class name and object center location are provided on the map. This human-readable map can be easily prepared by a person who can simply approximate the object centers’ locations by marking points on a 2D floor map. Because our method only operates using a small amount of information in an indoor environment, a large FOV is crucial for robustness against blocked views. Therefore, we used a spherical camera with a $360^{\circ }$ FOV. Depending on their size and use, the numbers of objects in the environment varied. Furthermore, the detection performance varied for all the object classes. Therefore, we evaluated our method on different object groups. To achieve a small, portable, low-cost system, we conducted our experiments with a person carrying a $360^{\circ }$ FOV camera and an IMU.

## 3. Sparse Semantic Localization

### 3.1. Approach

In this study, the problem of sequentially finding the 2D pose of the user on a 2D annotated floor map using sparse semantic information to achieve an efficient and robust indoor localization is addressed. The system overview is shown in Figure 1. The 2D annotated floor map was prepared in advance. Members of the predetermined object classes (such as windows and doors) were annotated on the 2D map without a unique ID. For each annotated object, only the 2D pose of the object center (x,y) and object class name were provided. The map did not convey any information on the physical appearances of the objects. The system consisted of a camera with a $360^{\circ }$ FOV and an IMU. As the user walked, the camera was used to obtain sparse semantic information from the user’s perspective through a trained CNN. The large FOV was used to obtain information from all directions. The sparse semantic information and IMU readings were sent to the sparse semantic localization system to update the user’s pose. In the following few sections, we will explain these steps.

### 3.2. Sparse Semantic Information

The semantic information must first be obtained from images before it can be used to perform localization. We only require the object class and relative bearing angle from the user’s perspective to the object center for each detected object. Because we did not perform image matching and only required the information listed above, we decided that pixel-wise segmentation and labeling were unnecessary for our system and thus opted to use a faster solution. YOLOv2 [24] is a real-time CNN that predicts the bounding boxes and probabilities for each image region, whereas Tiny YOLOv2 is a smaller model for constrained environments. Because our environment consists of predetermined objects, Tiny YOLOv2 was ideal for the object detection task. The user carried a spherical camera with $360^{\circ }$ FOV to obtain as much information about the environment as possible. The dual fish-eye images obtained from the spherical camera were converted into equirectangular images to make it more convenient to train a CNN and calculate the bearing angles to the object centers. Despite the distortions caused by the spherical camera, the CNN was trained using our equirectangular images dataset. The object classes chosen for our system were tables, boards, chairs, windows and doors. The CNN provided the object class and bounding box of each detected object. The relative bearing angles were calculated based on the bounding box centers.

**Figure 2:**
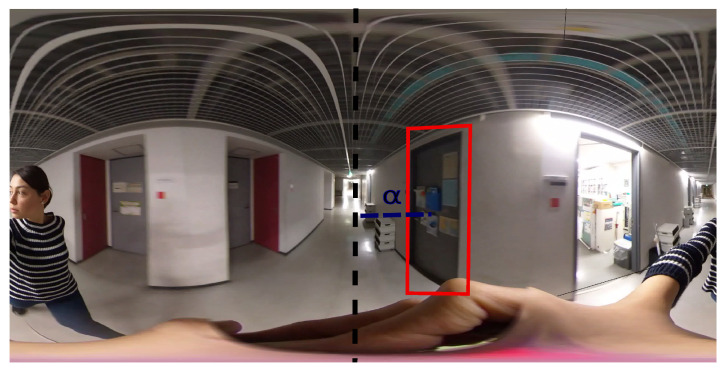
Example for visual input: The camera is held by the user. The middle of the equirectangular image is considered to be zero. The sample predetermined object class “door” has a detection result in the bounding box.

A representative image can be seen in Figure 2. If the center of the equirectangular image was the point faced by the user, the bearing angle to an object’s bounding box center was calculated by first converting the bounding box center pixel number into degrees, dividing the column number of the bounding box center by the image width and multiplying it by 360. Because the left top point of the image was the zero-degree point, to shift it to the image center, which was the zero point for the user, 180${}^{\circ }$ was added to the pixels that were on the left side of the centerline of the equirectangular image. If α was the bearing angle to the center of the object from the reference point faced by the user, the width was the column number of an equirectangular image, and $p_{x}$ was the column number of the pixel at the center of a bounding box. Then, α was calculated as

(1) $$\begin{array}{c} \alpha =\left\{\begin{array}{cc} (p_{x}/w)\times 360+180, & \mathrm{if}\;p_{x}<w/2\\ (p_{x}/w)\times 360-180, & \mathrm{otherwise} \end{array}\right. \end{array}$$

### 3.3. Localization

#### 3.3.1. Sensor Model

The MCL [25] approach was used for localization. MCL—a recursive Bayes filter for estimating the pose of a user in motion—senses the environment. The Bayes filters estimate the state of a dynamic system from the sensor measurements while assuming the Markov property of the environment. The current state’s probability distribution depends only on the previous state. Different sensors, such as LiDARs, range finders, or cameras, can be used for the MCL. Robots with range finders generally compare range and bearing angle information to a pre-built distance map. Each particle in the MCL represents a pose hypothesis and has a weight. The sum of the particles’ weights is one. As the user moves around and senses the environment, the particles or the pose hypothesis that do not correlate with the observations are eliminated.

The MCL has three stages: prediction, updating and resampling. MCL, without any prior information on the pose, generally starts with the uniform distribution of particles. Odometry information is used in the prediction step to propagate the particles. The prediction step is followed by an update step, where the propagated particles’ weights are updated proportionally to determine their degree of correlation with the current observations. Because localization is assumed to be a dynamic Bayesian network, only the latest sensor measurement and odometry is used. Finally, at the resampling stage, the particles are resampled based on their weights. Less likely particles are replaced by more likely ones, and the particles converge toward the user’s location.

In our method, the MCL required an annotated 2D map **M** that stored the Cartesian coordinates (x,y) of annotated objects from the different predetermined object classes *c*, state(x,y,θ) of the user **s**, odometry information **u** and the sensor update **Z**. The map **M** was defined as **M**$={\left\{x_{n},y_{n},c_{n}\right\}}_{n=1}^{N}$, where *N* was the number of objects in the map. The sensor measurements **Z** consisted of the detected object class *c* and bearing angle α to the center of the object. The measurement update **Z** was defined as **Z**$={\left\{\left(c_{k},\alpha_{k}\right)\right\}}_{k=1}^{K}$, where *K* was the number of objects in each sensor reading.

In the prediction step, all the particles from $P(s_{t-1}|z_{t-1},u_{t-1})$ were propagated using a motion model. Afterwards, in the update step, each particle weight was updated according to how well the current observations matched the map made by a sensor model $P(z_{t}|s_{t},M)$. Finally, a resampling method was used based on posteriors $P(s_{t}|z_{t},u_{t})$. The posterior is calculated as follows:

(2) $$\begin{array}{ccc} \; & P(s_{t}|Z_{t},u_{t},M)=\; & P\left(Z_{t}|s_{t},M\right)P\left(s_{t}|u_{t},s_{t-1}\right)P\left(s_{t-1}|Z_{t-1},u_{t-1},M\right) \end{array}$$

Generally, measurements are assumed to be conditionally independent of each other given their location; thus, the product of the likelihood is applied as follows:

(3) $$\begin{array}{c} P\left(\left\{z_{1},z_{2},\ldots ,z_{k}\right\}|s,M\right)=\prod_{k=1}^{K}P\left({\left\{z\right\}}_{i,k}|s,M\right) \end{array}$$

However, with this approach, the generated posterior distributions often result in overconfident estimations [26]. In this study, we assumed that there was a dependence within the object classes and independence across object classes. The sensor model included the class type of the observed object and the bearing angle to the center of the object on a 2D plane. Objects belonging to the same class were grouped as $O_{i}=\left\{Z_{k}:c_{k}=c_{i}\;\forall k\right\}$, where *C* was the number of object classes. The measurement probabilities were weighted by $P{\left(z|s,M\right)}^{1/size(O_{i})}$, the inverse of the number of objects of the same class present, to prevent the objects present in a larger number from dominating. Our model could be considered a variant of the general Product of Experts (PoE) proposed in [26], where the total sum of weights equals the number of classes and the weights for each class are fixed. In our case, it is applied within the same object class.

If $z_{p}$ and $z_{q}$ were two objects from the same class, we can represent $P(z_{p},z_{q}|s)$ as $P\left(z_{p}|s\right)P\left(z_{q}|z_{p},s\right)$ or $P\left(z_{q}|s\right)P\left(z_{p}|z_{q},s\right)$. Their multiplication would lead to

(4) $$\begin{array}{ccc} \; & P{\left(z_{p},z_{q}|s\right)}^{2}=\; & P\left(z_{p}|s\right)P\left(z_{q}|s\right)P\left(z_{q}|z_{p},s\right)P\left(z_{p}|z_{q},s\right) \end{array}$$

Based on the assumption of the complete dependence of these two measurements, this equation would result in

(5) $$\begin{array}{cc} \; & P\left(z_{p},z_{q}|s\right)=P{\left(z_{p}|s\right)}^{1/2}P{\left(z_{q}|s\right)}^{1/2} \end{array}$$

for two measurements, *p* and *q*, of the same class. If we generalize this to the *K* objects at a reading, we would obtain

(6) $$\begin{array}{c} P\left(\left\{O_{i}\right\}|s,M\right)=\prod_{k=1}^{K}P{\left({\left\{O\right\}}_{i,k}|s,M\right)}^{1/k} \end{array}$$

Applying the PoE model can solve the overconfidence issue by smoothing the joint likelihood. The assumption of dependency can cause underconfidence. However, in practice, underconfidence may yield better results than overconfidence. The particle weights were updated based on the maximum likelihood correspondence [27]. Because the objects in the map and in the sensor data were not uniquely identified, for each piece of observation data, the most likely map objects were selected to be true. Introducing semantic information provided distinctions among the objects, especially if the number of elements in an object class was low. The observations were matched as follows:

(7) $$\begin{array}{ccc} \; & P(z^{k}|s,M)=\; & \max(P\left(z^{k}|s,M^{n}\right)\forall M^{n}\in M|M_{c}^{n}=z_{c}^{k}) \end{array}$$

#### 3.3.2. Motion

In the prediction step of MCL, odometry information is required. Robots generally estimate their motion using various devices such as wheel encoders. To estimate the odometry for people, the IMU and visual odometry are among the feasible solutions. In our method, the camera carried by the user was a spherical camera. Although visual odometry methods have been developed for spherical cameras, they are limited compared to those employed for monocular cameras. Furthermore, they seem to have the tendency to lose the estimated visual features in plain environments dominated by white walls [28,29]. The motion of the user can also be calculated using an inexpensive and lightweight IMU. However, it is almost impossible to use the IMU data alone, because they suffer from noise, accumulating error and drift. To cope with these problems, a low-pass filter was coupled with a median filter to reduce the noise of the accelerometer. To reduce the error of the yaw angle, a complementary filter was used to integrate the accelerometer and gyroscope data [30].

## 4. Experiments

### 4.1. Environment

**Figure 3:**
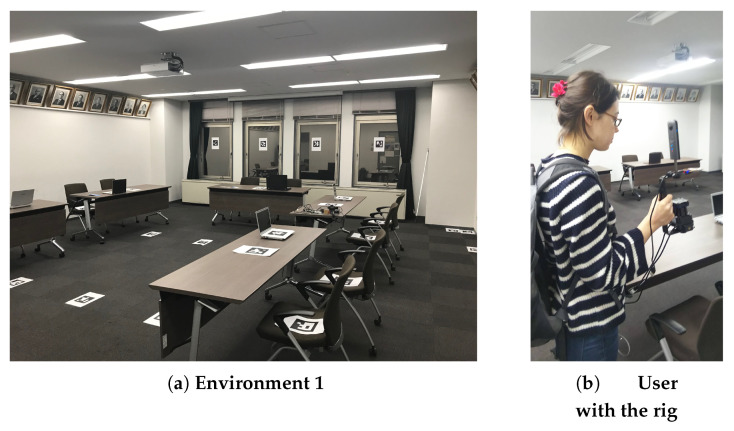
Room-Level Experiment Setting. (**a**) Experiment environment with ArUco markers. ArUco markers are placed on objects to annotate the 2D map. Extra markers are put on the floor to ensure continuity during marker capture for map creation. (**b**) User with experiment equipment, including a rig to hold the sensors required to capture sensor data for the experiment and ground truth for validation. The rig consists of a Ricoh Theta V camera, Sparkfun Razor inertial measurement unit (IMU) for system input and a Hokuyo range finder to construct the ground truth.

**Figure 4:**
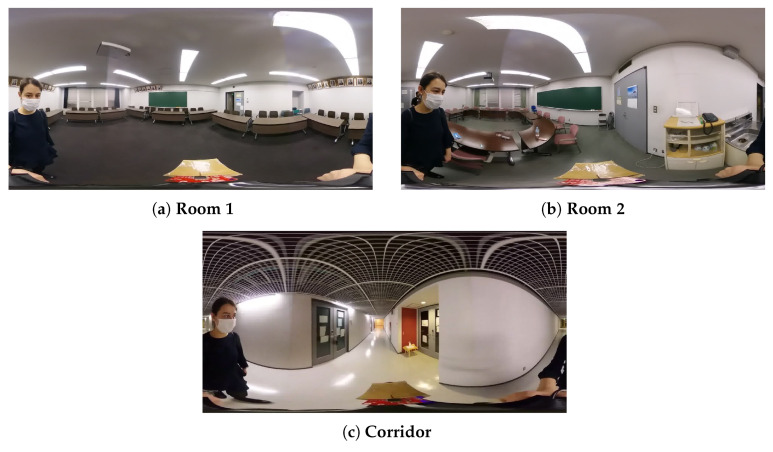
Symmetrical environment experiment setting. (**a**) Starting room, (**b**) final room, (**c**) connecting corridor.

The environment we chose for the room-level experiment was a classroom with a board, tables, chairs, windows, pictures and a door. A 2D annotated map was required for our method. This map was prepared before the experiment using ArUco markers to represent the objects and ArUco SLAM. ArUco markers are binary square fiducial markers for fast and straightforward camera pose estimation [31]. Each object and room corner was assigned a marker with a unique ID. The marker arrangement can be seen in Figure 3a. In the last experiment, two rooms with the same object classes and a corridor were used. The experimental setting can be seen in Figure 4. The annotated map was created manually without any sensor measurement.

### 4.2. Ground Truth

The use of motion trackers is one of the possible methods to obtain the ground truth. However, they constrain the user’s motion in further experiments. Therefore, we preferred to use a Hokuyo range finder with a cartographer [32]. The user carried the range finder during the experiment. The cartographer used the IMU, which was carried by the walking user for motion information. Then, the trajectory was extracted for use as the ground truth. According to [33], in which trajectories in an indoor office environment were compared, the cartographer had an error rate of up to 0.017±0.021 m, which is a sufficient level of accuracy for our task.

### 4.3. Sensors

For the vision module, a Ricoh Theta V camera was used to record images. It was used with a Tiny YOLOv2 trained on our equirectangular images for six object classes (door, window, table, laptop, chair, and board). For motion, Sparkfun 9DOF Razor IMU M0-SEN-14001 was used. The IMU was set up to face the same direction as the user. The camera was also placed such as to ensure the center of an equirectangular image was where the user faced. To obtain the ground truth, a rig was used to connect those three sensors (IMU and camera for the sensor model, and range finder for the ground truth) to the same frame. The rig is shown in Figure 3b.

### 4.4. Experimental Setting

We conducted different experiments to evaluate our sensor model both in a simulation and the real world. The experiments in the Unity simulation are detailed in our previous work [34]. In this section, we will focus on our experiments in the real world.

The room-level experiment’s 2D annotated map was created using ArUco markers and mapping [31]. The object information was obtained from the videos of the Ricoh Theta V camera carried by the user during the experiment. These images were first converted to equirectangular images offline. The objects of the predetermined object classes were detected in real-time using a Tiny YOLOv2 node. After the objects were detected, their bearing angles to the object centers were calculated. Then, the class name and bearing angle information were sent to the MCL node for localization. The IMU data were filtered before they were sent to the MCL. The experiments were conducted using 500 particles that were initialized randomly. The ground truth for the user trajectory was collected during the experiment. The ground truth trajectory points were matched with the poses that were calculated according to the timestamps of the IMU. The trajectory was surrounded by different types of objects. The length of the trajectory was 30.5 m. The semantic information in the experiment environment consisted of one door, four windows, one board, five tables, ten chairs, four laptops and several frames on the wall. All the objects, excluding the frames and laptops, were included in the 2D annotated map. For each experiment type, the compared perception models were run 30 times in parallel. The average result is shown in the tables and figures. In all tables, the result shown is the root mean squared error (RMSE) calculation after convergence.

In the experiments, a human—instead of a robot—was localized. The user walked in the environment at different speeds. The change in the speed affected the drift of the IMU data and caused motion blur in some images, especially at the corners. For the majority of the time, the user walked between objects and a wall while facing another room wall or a corner, resulting in a partially blocked view. Although a camera with a $360^{\circ }$ FOV was used in the experiments, it should be noted that a part of the image was consistently blocked by the user. Approximately $60^{\circ }$ (between $180^{\circ }$ and $240^{\circ }$) was blocked by the user at each frame. Therefore, the results from the experiments using a perception model with a $360^{\circ }$ FOV were from a lower FOV. As mentioned earlier, the localization system was evaluated according to its constraints. We conducted two types of experiments. First, to evaluate the robustness to the partial view obstruction caused by the user walking close to the walls and large objects in an indoor environment, experiments with $120^{\circ }$, $180^{\circ }$ and $360^{\circ }$ FOVs were compared for a trajectory with which the user walked close to the wall and objects. To evaluate the robustness of all the sensor models to map errors, their performance was evaluated using annotated maps with a different range of annotation errors. Second, to evaluate the effect and significance of the object class groups, which varied in terms of number and detection performance, perception models using different object groups were evaluated. In the third experiment, the system behavior on the same trajectory was evaluated in the presence of sensor error. In the last experiment, the performance of the system was evaluated for two similar rooms and a corridor. The experiment setting can be seen in Figure 4. In this experiment, chairs were not included in the annotations, and the 2D map annotations were made manually without ArUco markers or any measurement.

### 4.5. Results

#### 4.5.1. Robustness to Crude Maps and Partial View Blockages

**Figure 5:**
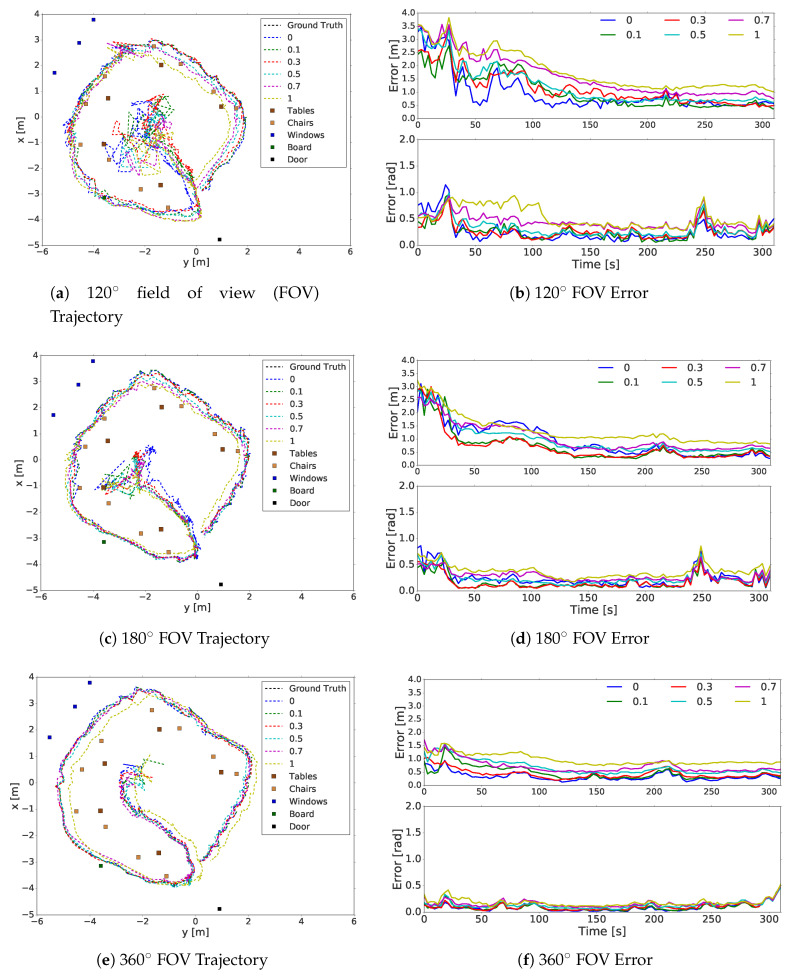
Trajectories and errors of sensor models with a $120^{\circ }$, $180^{\circ }$ and $360^{\circ }$ FOV. Figures (**a**,**c**,**e**) show the generated trajectory on maps with a noise of σ=0 (blue), σ=0.1 (green), σ=0.3 (red), σ=0.5 (turquoise), σ=0.7 (magenta) and σ=1 (yellow). The ground truth is shown in black. Figures (**b**,**d**,**f**) show the localization errors of corresponding trajectories.

The FOVs of the perception models used in the experiment were $120^{\circ }$, $180^{\circ }$ and $360^{\circ }$. They were calculated by taking the middle of the image as the zero points and positioning half of the desired FOV on the right and left sides of the images. The object classes used by the sensor model were the door, board, window, table and chair. Noise was added to the annotated objects on the 2D annotated map with standard deviations of σ=0.0, σ=0.1, σ=0.3, σ=0.5, σ=0.7 and σ=1.0 on the x and y-axis. In Figure 5, the trajectories and RMSE of the sensor models with a $120^{\circ }$ FOV, $180^{\circ }$ FOV and $360^{\circ }$ FOV are shown, respectively. In Figure 5a,c,e, the location of the object centers in the office (tables, windows, door, etc.) are indicated using markers in different colors. Table 1 shows the total localization error after the convergence point at timestep 50.

As can be seen from Table 1, the worst performance was yielded by the perception model with a $120^{\circ }$ FOV with 0.62±0.22 m and 0.24±0.22 rad errors, whereas the perception model with a $360^{\circ }$ FOV had the best performance among the perception models, with 0.36±0.15 m and 0.21±0.24 rad errors. As the FOV increased, the accuracy improved. The FOV also affected the convergence speed. A larger FOV corresponded to faster convergence. As expected, walking towards corners and turning were challenging for the localization system. Although a large FOV made the system robust to the blocked view problem, the sensor model with a $120^{\circ }$ FOV was affected, as is visible in Figure 5a. Adding generated noise with the given standard deviations increased the error, but the localization mostly proved to be robust to noise and approximations. Overall, the sensor model with a $360^{\circ }$ FOV had smoother trajectories compared to the other sensor models.

#### 4.5.2. Robustness to Different Object Classes

**Figure 6:**
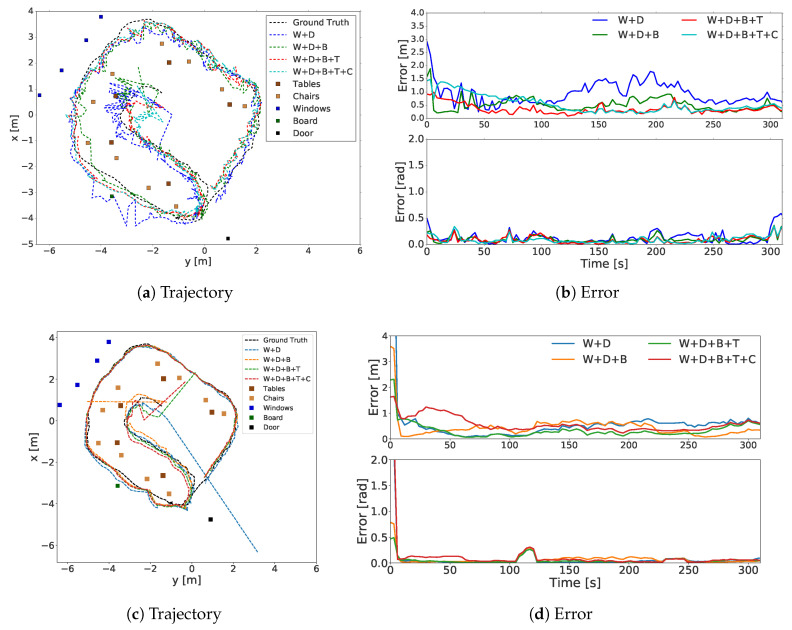
Trajectories and errors of sensor models using different object classes. Sensor models used different combinations of W (window), D (door), B (board), T (table) and C (chair) object classes. Subfigures (**a**,**b**) show the trajectory and errors of the sensor model using different object class combinations, respectively. Subfigures (**c**,**d**) show the trajectory and errors corresponding to the sensor model using the ground truth for object detections.

To evaluate the effect of different object classes in the environment, we performed experiments with perception models using different classes of detected objects in sensor updates. All the perception models used a $360^{\circ }$ FOV. In this experiment, the annotated map was noise-free. The first sensor model used only the window and door; the second model only considered the window (W), door (D), and board (B); and the third considered the table (T) in addition to the objects used in the second model. The fourth sensor model used the door, window, board, table and chair (C) object classes. These sensor models are represented with blue, green, red and turquoise, respectively, in Figure 6. The ground truth is shown in black. The average trajectories of the perception models are shown in Figure 6a. The objects in the environment are indicated with markers. The RMSE of the perception models is presented in Figure 6b, from the initialization time and in Table 2 after timestep 50. As shown in Table 2, the sensor models using the largest number of object classes yielded better results, with 0.38±0.16 m and 0.21±0.25, whereas the one that used the window and door classes yielded the worst result, with 0.68±0.30 m and 0.24±0.29 rad errors. Unlike the chair class, including the board and tables improved the result significantly.

To better assess the effect of each object class, we conducted an experiment in which, instead of Tiny YOLov2 detections, the bearing angles to the object centers were calculated from the ground truth positions obtained using the cartographer to obtain the true locations of the annotated objects on the annotated map. Additional data associations, such as object matching and observation IDs, were not considered. The trajectories and errors are presented in Figure 6c,d. As shown in Table 2, the sensor model that used the window and door classes yielded the worst result, with 0.40±0.18 m and 0.04±0.08 rad errors; the best results were yielded by the sensor model that considered the window, door, board and table object classes, with 0.21±0.14 m and 0.03±0.07 rad errors. As in the previous experiment, including the chair did not affect the result significantly.

#### 4.5.3. Relocalization

To evaluate the system behavior in the presence of sensor errors, we conducted an experiment in which the system was introduced to a sudden jump in its position. Three positions on the trajectory was selected. At each position—3 m, 5 m or 7 m—errors were manually introduced to a different particle filter separately, meaning that the recovery at each position at different range of error could be observed. The localization errors, shown in Table 3, were calculated when the system converged (at 200 s for the first and the second jump position, and after 220 s for the third jump position).

**Figure 7:**
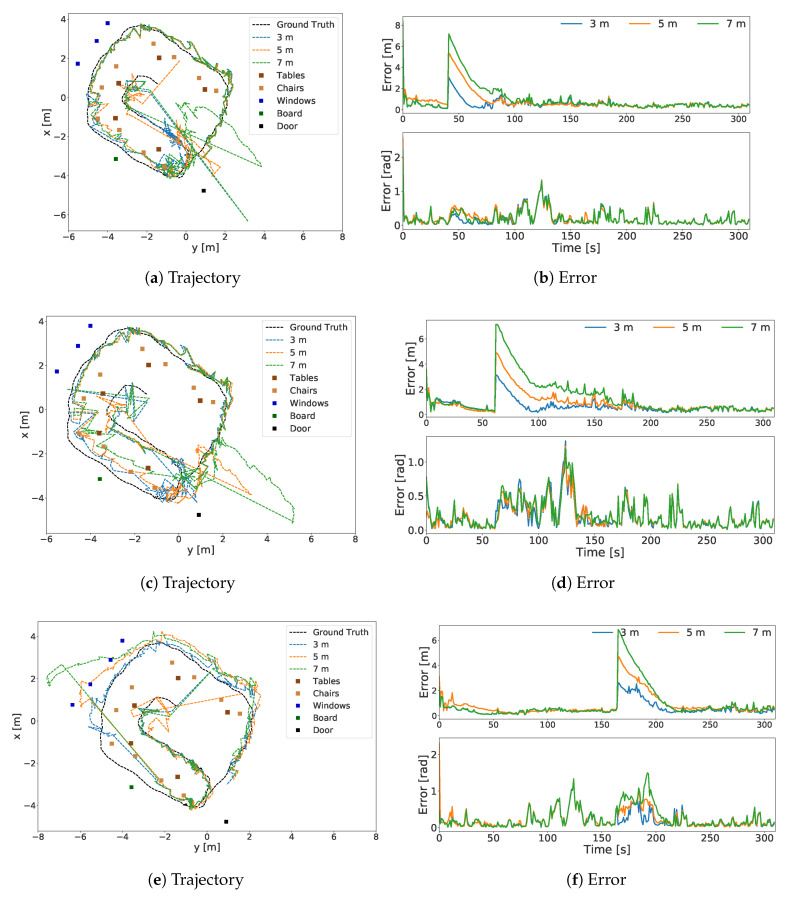
Sensor error: jump. Sensor models were exposed to 3 m, 5 m and 7 m jumps at three different timesteps. Subfigures (**a**,**b**) belong to the first jump. Subfigures (**c**,**d**) show the second jump. Subfigures (**e**,**f**) show the third jump.

The first localization error was introduced at a location at which the system had a good view of the room. After the jumps in the position, the particles were still close to the objects; therefore, the system managed to recover. The trajectories and errors for this case are shown in Figure 7a,b.

The second localization error was introduced at a location close to a room border. When introducing localization errors at 5 m and 7 m, the particles ended up being far behind the objects. In this experiment, we assumed this situation was a recovery case in a large room, where the objects are on the far left and concentrated on a single side. The trajectories and errors for this case are shown in Figure 7c,d. Although relocalization took longer than in the previous case, the system still converged to the correct trajectory. However, it should be noted that the system waited for half of the time between timesteps 100 and 150. Therefore, this situation affected the correction speed. The system could relocate itself on the trajectory with the same performance after a 3 m jump; however, the error increased for the cases of 5 m and 7 m. The errors in these cases increased to 0.43 ± 0.13 m with 0.12 ± 0.11 rad, and 0.41 ± 0.13 m with 0.12 ± 0.10 rad on average, respectively.

The third localization error was also introduced at a location close to a border. Therefore, it also experienced the same challenges as the second experiment. In addition, the door was blocked by the user, and the board could not be reliably detected due to the user’s proximity to it. The trajectories and errors for these cases are shown in Figure 7e,f. After the 3 m jump, the average localization error was 0.39 ± 0.13 m with 0.12 ± 0.11 rad, whereas the error increased to 0.56 ± 0.14 m with 0.12 ± 0.10 rad after the 5 m jump, and 0.53 ± 0.17 m 0.13 ± 0.10 rad after the 7 m jump.

#### 4.5.4. Symmetric Rooms

**Figure 8:**
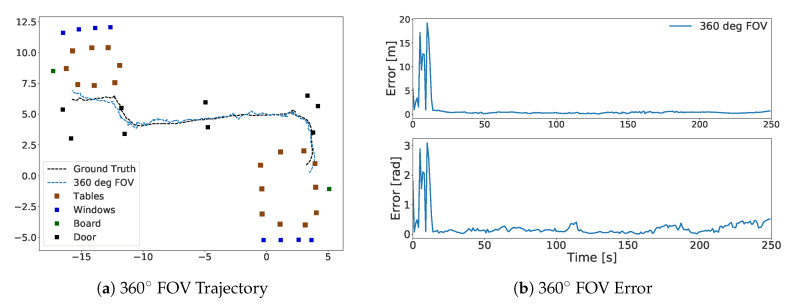
Trajectory and error for experiment in two rooms and corridor. Figure (**a**) shows the generated trajectory after convergence. (**b**) shows the error from initialization time. The ground truth is shown in black.

An experiment with two similar rooms and a corridor was conducted to evaluate the performance of our approach in a large scenario. The object classes used in this experiment were the window, door, board and table. Both rooms had objects from the board, door, window and table classes; the corridor had only objects from the door class. Doors were located on both sides of the corridor, with one at the end. As the subject walked in the corridor, the lowests number of objects visible to the camera was three. In this experiment, annotations were made without measurement or ArUco markers but by marking down the approximate object centers on the 2D map by looking at the photos of the environment. The object distribution was similar in both rooms, except that one room had an extra door and the other had two extra tables. Random global initialization was used to start the system. The particles were compared to the annotated objects that were at a maximum of 6 meters distance from them. If no window, table or board objects were detected, it was assumed that the system was located in the corridor. The biggest challenge in this experiment was whether the system identified the correct room. When the object weights were set as the inverse of the total number of objects in either room, the sensor model often confused the starting room. Considering the almost exact distributions of the two halves of the environment, except for two tables and a door, this situation was expected. To solve this, we prioritized the table class by setting its weight to 1, and that of the other object classes as the inverse of its instances in one of the rooms. Using these weights for all 30 runs, the sensor model correctly converged to the correct starting room. The trajectory is shown in Figure 8a, and errors are shown in Figure 8b. The trajectory in Figure 8a was drawn once the system converged, and the errors in Figure 8b are shown from the start. In Table 4, the root-mean-square error (RSME) is given. The average error was 0.38±0.17 m and 0.17±0.13 rad. This experiment emphasized the importance of learning the object class weights based on the environment.

#### 4.5.5. Efficiency

Although our method required a 2D floor map, in the update step of the localization method, the observations were compared using annotated objects represented only by their object centers (x,y) and object class names. During the localization process, the amount of information used at the update step was approximately 29 times smaller in our model than the range information when other methods, such as cartography, were used. The input to our sensor model was measured to be approximately 301 bytes, whereas the range readings were 8742 bytes per scan. On a machine equipped with an Intel Core i7-8700 CPU (3.20 GHz × 12) and a Geforce GTX 1070 GPU, the Tiny YOLOv2 node took 0.0117 s and the MCL node took 0.0165s. The performance of the MCL mostly depended on the particle number. We did not expect a great change in its performance in a lower system. However, the performance of the Tiny YOLOv2 is subject to the GPU power; therefore, we believe the bottleneck of our approach is the Tiny YOLOv2. Our system cannot be categorized as a system with limited resources. For this category, the Nvidia Jetson Nano is an ideal and popular option. Tiny YOLOv3 is a comparable CNN to Tiny YOLOv2 and has a frame rate of 25 fps on Jetson Nano according to the Jetson Nano benchmark (Jetson Nano: Deep Learning Inference Benchmarks, https://developer.nvidia.com/embedded/jetson-nano-dl-inference-benchmarks). Thus, we believe that it should be possible to obtain a reasonable result using a smaller system.

## 5. Discussion and Conclusions

In this paper, we presented a straightforward and efficient localization system that uses a small amount of sensor input to localize the user on a 2D map; the system is robust to a partial blockage of view and does not require highly accurate maps. Partially blocked views are a common problem in vision-based methods for localization indoors, because rooms mostly consist of white walls and corners, which are textureless. Therefore, a camera pointing at them cannot retrieve much information. A solution is to use a spherical camera, which can capture data from all directions. Therefore, even when the user walks beside one wall while facing another wall, the camera can capture useful information from the non-blocked sides. Robustness against errors in maps is a highly desirable characteristic, as it lowers the burden during map creation. If the localization method can work on approximately accurate maps, then regular users can create and update maps by hand, without sensors or complicated procedures. To evaluate the robustness of the proposed sensor model against partial blockage of views and map noise, we compared its performance using different FOVs for self-localization on a map with varying noise levels. Our experiments showed that a large FOV was crucial for accuracy, convergence speed and robustness against blocked views and map noise.

Our method is effective for with different object classes, which have different effects on the localization result, depending on their different shapes, numbers and sizes. To evaluate the impact of different object classes, sensor models using different object classes were compared in two tests. The first test detected objects using Tiny YOLOv2. Therefore, the detection errors for each class also played a role in the outputs. The second test replaced Tiny YOLOv2 with the annotations from the 2D map directly. Thus, they were not affected by the Tiny YOLOv2 errors. The object classes considered by the sensor models ranged from only a few classes, such as windows and doors, to all object classes. Other than the effect of the different classes, these experiments showed that a significant portion of the localization errors was caused by detection errors. Here, we include our observations for the different object classes.

Doors are an essential part of any building. Except in corridors, they do not exist in large numbers, which reduces the entropy in poses when they are observed. However, as most rooms in a building have doors in similar locations, they are not effective at reducing uncertainty in larger experiments. In the first two experiments, between 160–190 s, when the user was walking in front of the board, the door was blocked by the user, which affected the system and created peaks in the trajectory error, as shown in Figure 6a, which also shows the trajectory when only the door and window classes were used. Other than in this case, the doors were visible during a large part of the trajectory and remained sufficiently large to be detected, even when the user walked far away from the doors. Therefore, we concluded that the door was a very reliable class that could have a large effect on the localization performance. Furthermore, in the corridor, the doors were the only visible objects, making them essential. Owing to the large FOV, although the corridor was not very wide, at least three doors were visible at all times, enabling localization.

Similar to the door class, the window class is part of the construction of the building. Therefore, it is a common object and an important class. In our experiment, the windows were located on the same wall, with a small distance between them. They all had light-gray blinds. Therefore, they were not very easy to detect in equirectangular images. As previously mentioned, during the second experiment, there was a noticeable increase in the localization error for the models using only the window and door classes between 160–190s. During this time, the door was blocked by the user, making the windows the only detected objects. As can be seen, the windows alone were not sufficient to keep the localization errors minimal.

The board class is generally large, and boards are found in small numbers, especially in classroom environments. Therefore, this class was easy to detect and match most of the time and very useful for efficiently reducing uncertainty. However, the board in the environment was wide, and when the user walked in front of it between 150–190s, the system suffered from bounding box jitter due to the partial detection in equirectangular images. The error is visible in Figure 6b for the sensor model using the window, door and object classes. Unlike the door class, the board class was only reliable from a certain distance. Despite this problem, its inclusion in the second experiment increased accuracy significantly.

In our experiment environment, there were many tables compared to the numbers of objects in other classes. Because they were sufficiently large, they were detected most of the time; however, they were not detected as reliably as the previously mentioned object classes. Because they existed in a large number in a symmetrical environment, there was also a data association problem, which became more difficult to handle with incorrect detection. At some locations, two tables had the appearance of a single object. The table class was also more affected by motion blur compared to the other object classes. This situation probably occurred due to the similar color of the tables and the ground in our experiment environment and the difficulty of distinguishing individual tables. In the second experiment, including the table class increased the accuracy significantly for the sensor model using the Tiny YOLOv2 detections. The previously mentioned bounding box jitter of the board, mismatch of the windows and partial blockage of the door problems were mostly compensated by the inclusion of the table class. In the third experiment, giving more weight to the tables broke the symmetry for the system. Unlike doors, windows, and boards, which are distributed more symmetrically, table locations varied more. We believe that, along with the window class, if we can correctly estimate when and where detection was more likely to fail, the addition of this class can increase the system performance considerably. However, this is left for future work.

There were many chairs in the experiment setting. Owing to their small size and position behind tables, they were more difficult to detect compared to the previously mentioned object classes. In many frames, they were misclassified as laptops or were not detected at all. The accuracy of our system was not improved by this class. We did not examine any relations between the object classes. However, the table and chair classes are closely related; they often almost always coexist. We leave it for future work to define these relations to take advantage of this class.

A localization system is prone to sensor failures in general. To test the robustness of our method against localization errors, we conducted a small experiment to see how well the system could recover from a sudden localization error. We selected three positions on our map and introduced 3 m, 5 m and 7 m localization errors at each position to see how well the system could recover from these. The results showed that, in the presence of surrounding objects, the system could successfully recover from these errors. However, it was more difficult to fully recover from large errors as the particles were pushed to a place in which the objects were concentrated on a single side.

To understand the system behavior, the previous experiments were conducted at the room level. However, we believe our system could also be of use in larger environments. Localization in a large environment introduces new challenges. The biggest challenge is to break symmetries and identify the correct room. If there is an object class which uniquely exists in a particular room, identification would be easy; however, in most buildings, rooms are almost identical and consist of the same object classes. In such a case, identification is nontrivial. To assess our system’s performance in such environments, we conducted an experiment on two almost identical rooms connected by a corridor. Furthermore, to validate our claim that our system did not require highly accurate annotated maps, we used a map that was annotated manually. There were very few differences between both rooms: one had two extra tables, and the other had one extra door. Setting the initial weights of the classes based on the number of object class members in each room often caused the system to misidentify the room. However, giving more weight to the classes that broke the symmetry in the environment enabled the system to identify the correct room. This shows the importance of learning weights according to the characteristics of the environment as a whole, rather than of individual rooms. Learning the adequate weights in this context is left for future work.

In addition to setting the object class relations and learning weights, more object classes, such as walls and corners, could be included to ensure sensor measurements in all indoor environments. One of our aims was to create a human-friendly localization system. Therefore, our localization algorithm works with a 2D annotated map, which can be obtained by marking approximate object centers on a blueprint by any person. MCL for human-friendly maps has been researched on a hand-drawn map in [21,35]. Unlike these methods, we only assumed human involvement in marking object centers on an already available 2D map and measured the performance in the real world by increasing the map noise for annotated objects systematically.

In this work, a robust and efficient sparse semantic indoor localization method using a human-readable map was achieved. Our approach relied on the distinction power of semantic information and used sparse semantic information for efficient indoor localization. It was proven to be robust to partial view blockage and maps with noise through experiments conducted using lightweight and low-cost sensors. Users can easily prepare the required annotated map using human-level information. Thus, we believe this localization system is particularly suitable for small indoor systems with limited resources.

## Figures and Tables

**Table 1:** Localization error: Sensor models with different FOVs ($120^{\circ }$, $180^{\circ }$, $360^{\circ }$) and different object groups (W: windows, D: door, B: board, All: window, door, board, tables and chairs).

Localization Error						
Map Noise	$120^{\circ }$ FOV		$180^{\circ }$ FOV		$360^{\circ }$ FOV	
σ	Error [m]	Error [rad]	Error [m]	Error [rad]	Error [m]	Error [rad]
0	0.62 ± 0.22	0.24 ± 0.22	0.49 ± 0.20	0.23 ± 0.23	0.36 ± 0.15	0.21 ± 0.24
0.1	0.67 ± 0.23	0.24 ± 0.22	0.47 ± 0.17	0.23 ± 0.22	0.38 ± 0.14	0.21 ± 0.24
0.3	0.72 ± 0.22	0.25 ± 0.22	0.52 ± 0.17	0.23 ± 0.23	0.45 ± 0.13	0.21 ± 0.23
0.5	0.87 ± 0.18	0.28 ± 0.20	0.66 ± 0.14	0.26 ± 0.22	0.67 ± 0.12	0.24 ± 0.21
0.7	1.05 ± 0.17	0.32 ± 0.19	0.83 ± 0.13	0.28 ± 0.19	0.74 ± 0.12	0.26 ± 0.21
1	1.32 ± 0.14	0.36 ± 0.18	1.09 ± 0.14	0.33 ± 0.18	1.21 ± 0.18	0.27 ± 0.21

**Table 2:** Localization error of perception models using different object classes: W: windows, D: door, B: board, All: window, door, board, tables and chairs.

Localization Error of Different Object Classes				
Perception Models	W+D	W+D+B	W+D+B+T	W+D+B+T+C
Error (Tiny YOLO) [m]	0.68 ± 0.30	0.50 ± 0.19	0.38 ± 0.16	0.37 ± 0.15
Error (Tiny YOLO) [rad]	0.24 ± 0.29	0.21 ± 0.25	0.21 ± 0.25	0.21 ± 0.25
Error (Annotations) [m]	0.40 ± 0.18	0.26 ± 0.16	0.21 ± 0.14	0.22 ± 0.15
Error (Annotations) [rad]	0.04 ± 0.08	0.04 ± 0.08	0.03 ± 0.07	0.04 ± 0.07

**Table 3:** Localization error: Sensor model errors that had a jump in their position at three different timesteps in three different ranges.

Localization Error After Jump in Position						
	1st Position		2nd Position		3rd Position	
Range	Error [m]	Error [rad]	Error [m]	Error [rad]	Error [m]	Error [rad]
3 m	0.37 ± 0.14	0.12 ± 0.11	0.38 ± 0.14	0.12 ± 0.11	0.39 ± 0.13	0.12 ± 0.11
5 m	0.36 ± 0.14	0.12 ± 0.11	0.43 ± 0.13	0.12 ± 0.11	0.56 ± 0.14	0.12 ± 0.10
7 m	0.37 ± 0.13	0.12 ± 0.11	0.41 ± 0.13	0.12 ± 0.11	0.53 ± 0.17	0.13 ± 0.10

**Table 4:** Localization error: symmetric rooms.

Localization Error: Symmetric Rooms	
Sensor Model	$360^{\circ }$ FOV with W+D+B+T
Error [m]	0.38 ± 0.17
Error [rad]	0.17 ± 0.13

## Abbreviations

The following abbreviations are used in this manuscript:

MCL	Monte Carlo Localization
IMU	Inertial Measurement Unit
FOV	Field of View
